# Management of Psychotic Symptoms in a Patient With Parkinson’s Disease Maintained on Levodopa-Carbidopa Intestinal Gel by Paliperidone Long-Acting Injection: A Case Report

**DOI:** 10.7759/cureus.21491

**Published:** 2022-01-22

**Authors:** Abdulkarim O Alanazi, Abdulaziz A Boqaeid, Mohammed A Alnuwaysir

**Affiliations:** 1 Medicine, King Saud Bin Abdulaziz University for Health Sciences College of Medicine, Riyadh, SAU; 2 Psychiatry, Prince Sultan Military Medical City, Riyadh, SAU

**Keywords:** paliperidone lai, psychosis, lcig, levodopa-carbidopa intestinal gel, parkinson disease

## Abstract

This case presents a 47-year-old man, without known past psychiatric history who developed psychotic symptoms including delusions of infidelity and had homicidal plans against his wife after 10 months of Levodopa-Carbidopa intestinal gel insertion (LCIG). The patient was diagnosed with Parkinson’s disease at age 34, which is being managed with LCIG. Patient Parkinson's symptoms were not well controlled with other pharmacological and surgical interventions tried previously. Despite the current guidelines in treating Parkinson’s disease psychosis, the treating teams have faced many difficulties with the management of this patient’s psychotic symptoms. After trying Risperidone Consta on August 24, 2018, the patient improved gradually, then he was shifted to Paliperidone long-acting injection (LAI) on September 12, 2018. One month later, the patient was seen in the outpatient department with much improvement in Paliperidone LAI. Reporting this case as the patient was seen on November 29, 2021, the patient is stable and doing well overall in terms of absent psychotic symptoms with minimal resting tremors. The success story of using LAIs such as our patient’s response to Paliperidone LAI can help other psychiatrists expand their treatment options when facing such difficulties.

## Introduction

Parkinson’s disease (PD) is a progressive degenerative neurological disorder with characteristic clinical findings including resting tremor, bradykinesia, rigidity, abnormal balance, gait, and posture [1]. The incidence of PD rises with age, significantly increases beyond the age of 60 with a rare incidence before the age of 40 [2]. There is no definitive cure for PD, but the management includes pharmacological and surgical interventions, which in turn helps in managing symptoms and improving quality of life by replacing the deficient dopamine in the central nervous system [3]. Effective agents that are considered for initial therapy for PD include levodopa, dopamine agonists, amantadine, selective monoamine oxidase B (MAO-B) inhibitors, and anticholinergic agents [3]. In advanced cases of PD, device-assisted therapy options include deep brain stimulation device (DBS), continuous subcutaneous apomorphine infusion (CSAI), and Levodopa-Carbidopa intestinal gel (LCIG) infusion should be considered [4]. Aside from the cardinal motor symptoms, PD may be associated with a wide range of non-motor symptoms, including cognitive impairment and neuropsychiatric disorders [5]. Second-generation antipsychotics are typically thought to be safer in PD patients due to decreased D2 antagonism, but they can also produce extrapyramidal symptoms, albeit at a lower rate than first-generation antipsychotics [6]. In the case presented in this report, the authors observed a patient with early-onset PD who developed psychotic features after LCIG insertion and was successfully treated with Paliperidone LAI with no complaints of side effects.

## Case presentation

The case that we are reporting is of a 47-year-old man, married with four children, and a medically retired soldier. He was diagnosed with early-onset PD at the age of 34. His illness was severely progressive and he had tried multiple pharmacological and surgical interventions including L-dopa and DBS. Eventually, the patient's condition was only slightly improving with LCIG insertion. After 10 months of insertion, the patient was presented for the first time to the psychiatry service in the emergency department as he was referred for assessment of suspiciousness and aggressive behavior for one week. On June 1, 2018, the patient presented to Prince Sultan Military Medical City, Riyadh, Saudi Arabia, with psychotic symptoms including delusions of infidelity, and had homicidal plans against his wife. The development of his psychotic symptoms was most likely related to LCIG insertion and self-controlling of dopamine infusion. Due to those severe symptoms, he was admitted to the psychiatric ward. He was then managed according to the available guidelines. In the beginning, a trial of Quetiapine was initiated reaching 800 mg with no improvement apart from being heavily sedated. On June 7, 2018, a clozapine pretreatment workup was established and Clozapine treatment was initiated. Clozapine was titrated gradually up to 250 mg/day. However, due to poor adherence and lack of insight, the patient stopped his medication and was not compliant with the investigations, so he was readmitted again soon after his discharge for the same delusions and safety concerns. To consider shifting to paliperidone, clozapine was stopped on August 7, 2018, and risperidone was given instead as a syrup reaching up to 1.5 mL, then Risperidone Consta 25 mg was given on August 24, 2018 every two weeks. As the patient showed mild improvement, he was discharged from the inpatient services on September 3, 2018 and continued his follow-ups in our Consultation-Liaison Psychiatry Out-Patient Department. Finally, a trial of Paliperidone LAI was initiated on September 12, 2018 following the switching strategy from Risperidone Consta to Paliperidone Palmitate 50 mg Intramuscular (IM) monthly dose. one month later, The patient was seen in the outpatient department showing much improvement on that dose. Reporting this case as the patient was seen on November 29, 2021, the patient is stable and doing well overall in terms of absent psychotic symptoms, minimal resting tremors thanks to the LCIG, and great social function that he got back together with his wife and reunited with his family.

## Discussion

The incidence of psychosis in PD ranges from 3% to 30% [7,8]. Visual hallucination is the most common reported psychotic symptom, followed by auditory hallucination, illusions, and paranoid ideation [7]. Long regarded to be simply side effects of dopaminergic therapies, psychotic symptoms are now considered to be a result of intricate interactions between disease and treatment-related effects [9]. The issue in using LCIG is that the patient can adjust the doses up to a higher level. In this regard, the literature showed two different reports about three patients with PD on LCIG who developed psychotic features after self-administering higher LCIG doses than recommended without consultation [10,11]. These previous findings go in line with our case, in which the cause of psychosis might be due to the dopaminergic effect. As those patients’ on LCIG have mostly reached a more severe level of disabling motor symptoms, it was not a viable option to discontinue the treatment with LCIG all at once. Also, due to the severity of the psychotic symptoms and the safety concerns at stake here, the patient’s psychotic symptoms had to be managed vigorously.

Clozapine and Pimavanserin are currently the recommended drugs for PD psychosis (PDP) [12], being the only two drugs having efficacy with minimal effect on motor function as suggested by meta-analysis [13]. Quetiapine, which is structurally similar to clozapine, produced equivocal outcomes regarding safety and efficacy in the treatment of psychosis in PD patients [14]. Regarding Risperidone, many physicians avoid considering it in PD patients because they believe it is poorly tolerated and may aggravate motor symptoms [15]. Ellis et al. found equivalent efficacy of the two antipsychotics in lowering psychosis in PD in an only double-blind, randomized trial of clozapine and risperidone in the treatment of psychosis, but risperidone showed a higher tendency to aggravate extrapyramidal symptoms [16]. Clozapine and quetiapine both may induce side effects, such as sedation and somnolence which may reduce everyday activity and increase the risk of falling [17]. In addition, clozapine-inducible agranulocytosis necessitates regular blood-cell monitoring, which limits its therapeutic utility [18].

Despite the current guidelines in treating PDP, the treating teams have faced many difficulties with the management of this patient’s psychotic symptoms. The patient shows no improvement besides being sedated with Quetiapine use and was not compliant to the regular white-blood-cell monitoring after Clozapine. Moreover, one of the reasons for such a poor response, in this case, is that the patient was controlling how much dopamine he was infusing which might have made his psychotic symptoms worse.

Paliperidone LAI, the primary active metabolite of risperidone, is considered an atypical antipsychotic [19]. Due to the issue of noncompliance, long-acting injectable antipsychotics (LAIAs) were developed to be taken only once every two to four weeks [19]. When compared to oral medication, LAIAs result in more constant blood levels of the drug and fewer dose-related adverse effects [19]. Paliperidone LAI revealed a low incidence of extrapyramidal symptoms (EPS)-related side effects [19]. Even though Paliperidone LAI may be a therapy option for PDP, there have been no published reports on Paliperidone LAI usage for PDP to the best of our knowledge. Overall, this case we are reporting represent uniqueness in many ways of which to consider; early-onset PD, multiple treatment trials including surgical intervention like DBS and LCIG, possibly leading to the patient’s current presentation with psychotic symptoms, the poor response to the commonly tolerated antipsychotics options, and the unusual trial of LAIs such as Paliperidone that eventually yielded a good outcome. Also, one of the many limitations we faced in managing this patient serious psychotic symptoms was establishing a baseline cognitive function assessment that might be impacted on the short or long-term use of antipsychotics or even PD course and prognosis.

## Conclusions

The complexity of this case that we are reporting is intriguing. In many ways, it shows that the current medical practices might enhance our understanding of how the psychiatric symptoms might emerge in the context of other medical conditions or their treatment. Early-onset Parkinson's disease and advanced treatment options come with a price to consider. The use of LAIs in those who are medically ill is worth studying even more. The success story of using LAIs such as our patient’s response to Paliperidone LAI can help other psychiatrists to expand their treatment options when facing such difficulties.
